# Gut microbiota-dependent mechanisms and efficacy of natural polysaccharides in multitarget antidepressant therapy: a systematic review

**DOI:** 10.3389/fpsyt.2026.1789107

**Published:** 2026-06-29

**Authors:** Haoqi Liu, Min Wang, Chen Bai, Jingchun Li, Kaiqiang Dong, Rongjuan Guo

**Affiliations:** 1 Beijing University of Chinese Medicine, Beijing, China; 2 The First Affiliated Hospital of Henan University of Chinese Medicine, Zhengzhou, China; 3 The Second Clinical Medical College of Beijing University of Chinese Medicine, Beijing, China

**Keywords:** depression, gut microbiota, microbiota-gut-brain axis, natural polysaccharides, systematic review

## Abstract

**Background:**

Depression is a mental disorder with emotional, physiological, and behavioral symptoms. Natural polysaccharides have attracted attention for their low toxicity and potential to improve depression, possibly through modulation of gut microbiota and related metabolic pathways. This systematic review aimed to summarize preclinical evidence on the antidepressant efficacy of natural polysaccharides and explore putative mechanisms involving the microbiota-gut-brain axis.

**Methods:**

Following Preferred Reporting Items for Systematic reviews and Meta-Analyses (PRISMA) guidelines, a comprehensive literature search was performed in PubMed, Web of Science, and Embase for studies up to October 4, 2025.

**Results:**

Twenty preclinical studies met the inclusion criteria. Polysaccharide treatment was generally associated with improved depression-like behaviors, normalized neurotransmitter levels, and reduced inflammation and oxidative stress. These effects were accompanied by changes in intestinal barrier function, gut microbiota composition, and short-chain fatty acid metabolism. However, substantial heterogeneity and a high risk of bias across the included studies limit causal interpretation.

**Conclusion:**

Natural polysaccharides show potential antidepressant effects in preclinical models, but the current evidence is preliminary. Well-designed translational and clinical studies are needed to advance these findings.

**Systematic review registration:**

https://www.crd.york.ac.uk/PROSPERO/view/CRD420261284093, identifier PROSPERO, CRD420261284093.

## Highlights

- Multiple mechanisms of action of natural polysaccharides against depression are summarized.
- Preclinical evidence supports the therapeutic potential of natural polysaccharides for depression.

## 1 Introduction

Major depressive disorder (MDD) represents a critical public health challenge, affecting over 300 million individuals globally (1, 2). Its primary manifestations include persistent anhedonia, cognitive decline, and physical impairment, rendering it a leading cause of disability (3, 4). Besides its well-known psychological symptoms, depression is associated with numerous physical health issues (5, 6). These systemic complications encompass the deterioration of the intestinal barrier, dysregulation of the neuroimmune system, and the progression of neurodegenerative changes (7, 8). Studies indicate that the combination of antidepressants and psychotherapy constitutes an effective treatment. Nevertheless, the advantages of this method are typically constrained, with most patients experiencing only moderate relief (9, 10). Significant individual variability exists in patients’ responses to treatment (11, 12). The pathophysiology of depression results from the interplay of multiple biological systems (13), including monoamine signaling, the hypothalamic-pituitary-adrenal (HPA) axis, neuroinflammation, and the gut-brain axis. Consequently, therapeutic strategies that focus solely on a single pathway are often insufficient (14, 15). The prolonged administration of antidepressant medications presents various health risks, particularly metabolic dysregulation and multiorgan toxicity (16). Consequently, research interest is growing in natural polysaccharides (17).

Natural polysaccharides are a distinct type of organic compound. For centuries, they have been integral to traditional medicine and diets, esteemed for promoting health and treating ailments (18, 19). Polysaccharides are large molecules composed of ten or more monosaccharide units (20). These units are connected by glycosidic bonds and can be arranged in straight chains or branched structures (21). Their advantageous safety profile, low toxicity, and diverse biological activities render them particularly attractive (22, 23). Current preclinical evidence suggests that natural polysaccharides are associated with multidimensional improvements in animal models of depression. At the behavioral level, polysaccharide treatment has been reported to decrease immobility time in forced swimming and tail suspension tests, and to increase sucrose preference and exploratory activity (24, 25). At the neurochemical level, changes such as restored monoamine neurotransmitter levels and receptor expression have been observed (26). Regarding inflammation, polysaccharides have been shown to reduce pro-inflammatory cytokine expression, potentially via inhibition of microglial activation (27). In the context of oxidative stress, increased superoxide dismutase (SOD) activity and decreased malondialdehyde (MDA) levels have been described (28). Taken together, these observations point to possible mechanisms involving behavioral-neurotransmitter and inflammation-oxidative stress pathways that may underlie the antidepressant-like effects of natural polysaccharides.

Moreover, evidence suggests that these compounds may help maintain gut barrier integrity and promote a resilient gut ecosystem (29). They have been reported to enhance the production of short-chain fatty acids (SCFAs) such as butyrate, acetate, and propionate (30, 31). Several studies have found that polysaccharide treatment is associated with reduced depressive-like behaviors in animal models, together with changes in gut microbiota composition, barrier function, and SCFA levels (26). It has been proposed that these microbial metabolites contribute to gut barrier integrity, systemic inflammation control, and neurotransmitter regulation, which are thought to be relevant to mental health (32). Nevertheless, current evidence is largely correlational, and associative mechanistic hypotheses remain to be suggested.

Numerous pharmacological studies have suggested the antidepressant effects of natural polysaccharides. However, a comprehensive overview of this field is still lacking. To address this knowledge gap, we compiled preclinical studies that investigated polysaccharide interventions in depression models. This study aimed to explore potential mechanisms underlying the antidepressant effects of polysaccharides, focusing on their regulation of gut microbiota and microbial metabolites. The synthesis of these preclinical behavioral and mechanistic insights may help guide the process of clinical translation.

## 2 Materials and methods

### 2.1 Search strategy and terms used

A comprehensive electronic search was performed in PubMed, Web of Science, and Embase to identify relevant experimental studies published up to October 4, 2025. The search strategy was developed by two independent reviewers (HQ-L and C-B) using MeSH terms and free-text words related to “depression”, “polysaccharides”, and “gut microbiota”. The complete search strings for each database are provided in **Supplementary Table**. The searches were executed independently by two reviewers (HQ-L and M-W).

### 2.2 Inclusion and exclusion criteria

The inclusion criteria were as follows: (1) *in vivo* studies utilizing animal experiments; (2) use of depression animal models; (3) intervention with natural polysaccharides as a single agent (no combination with other active compounds or herbal extracts); (4) assessment of at least one depression-like behavioral outcome and one gut microbiota-related outcome. Measurement of neuroinflammatory markers or monoamine neurotransmitters was considered supportive but not mandatory for inclusion.

The exclusion criteria were as follows: (1) studies, including *in vitro* studies, case reports, clinical trials, reviews, abstracts, or comments; (2) studies that involved combination treatments with other compounds or herbal products; (3) studies that do not utilize depression models; (4) absence of high-throughput gut microbiota profiling or failure to report microbiota-metabolite correlation analyses.

### 2.3 Study selection

This study employed a triple independent screening mechanism executed collaboratively by three researchers (HQ-L, B-C, and M-W) for data selection. The literature processing comprised three sequential phases: (1) Duplicate removal based on metadata (PMID, title, authors, publication year); (2) title/abstract screening to systematically exclude reviews (including systematic reviews and meta-analyses), commentaries, and observational clinical studies (prospective and retrospective designs); (3) independent full-text review and data extraction by two investigators (HQ-L and M-W), with simultaneous cross-referencing of citations to identified potentially relevant literature. For unavailable full texts, corresponding authors were contacted via email for supplementation, and non-responses within two weeks were classified as invalid data exclusion.

### 2.4 Extraction and analysis

The extraction included the following domains: bibliographic data (first author and publication year), polysaccharide attributes (nomenclature, botanical origin, medicinal part, chemical composition, and molecular weight), experimental subject details (animal strain, body weight, age, sex, and sample size), experimental intervention parameters (modeling methodology, treatment protocol, dosage, administration route, and intervention duration), and outcome measures (behavioral improvement indicators, neurobiochemical indicators, neuroinflammatory indicators, oxidative stress indicators, intestinal barrier integrity, microbial community characteristics, and microbial metabolites).

### 2.5 Risk-of-bias assessment

To improve the scientific rigor and reliability of the systematic review, the risk of bias in the included studies was carefully evaluated using the Systematic Review Center for Laboratory Animal Experimentation’s Risk of Bias Tool (SYRCLE’s RoB tool) (33). Derived from the Cochrane Risk of Bias Tool for randomized controlled trials, SYRCLE’s RoB tool is specifically designed to evaluate the internal validity of animal studies (34, 35). This tool comprises six domains and ten items: (1) selection bias (sequence generation, baseline characteristics, and allocation concealment); (2) performance bias (random housing and blinding); (3) detection bias (random outcome assessment and blinding); (4) attrition bias (incomplete outcome data); (5) reporting bias (selective outcome reporting); (6) other biases (other potential sources of bias). Each entry was evaluated according to a three-tiered classification: “+” represented a low risk of bias, “-” denoted a high risk of bias, and “?” represented unclear risk due to inadequate reporting. A point was exclusively granted for items designated as “+, ” with no points given for “-” or “?” judgments. Two reviewers (HQ-L and M-W) independently conducted all assessments.

## 3 Results

### 3.1 Literature selection

**Figure 1:**
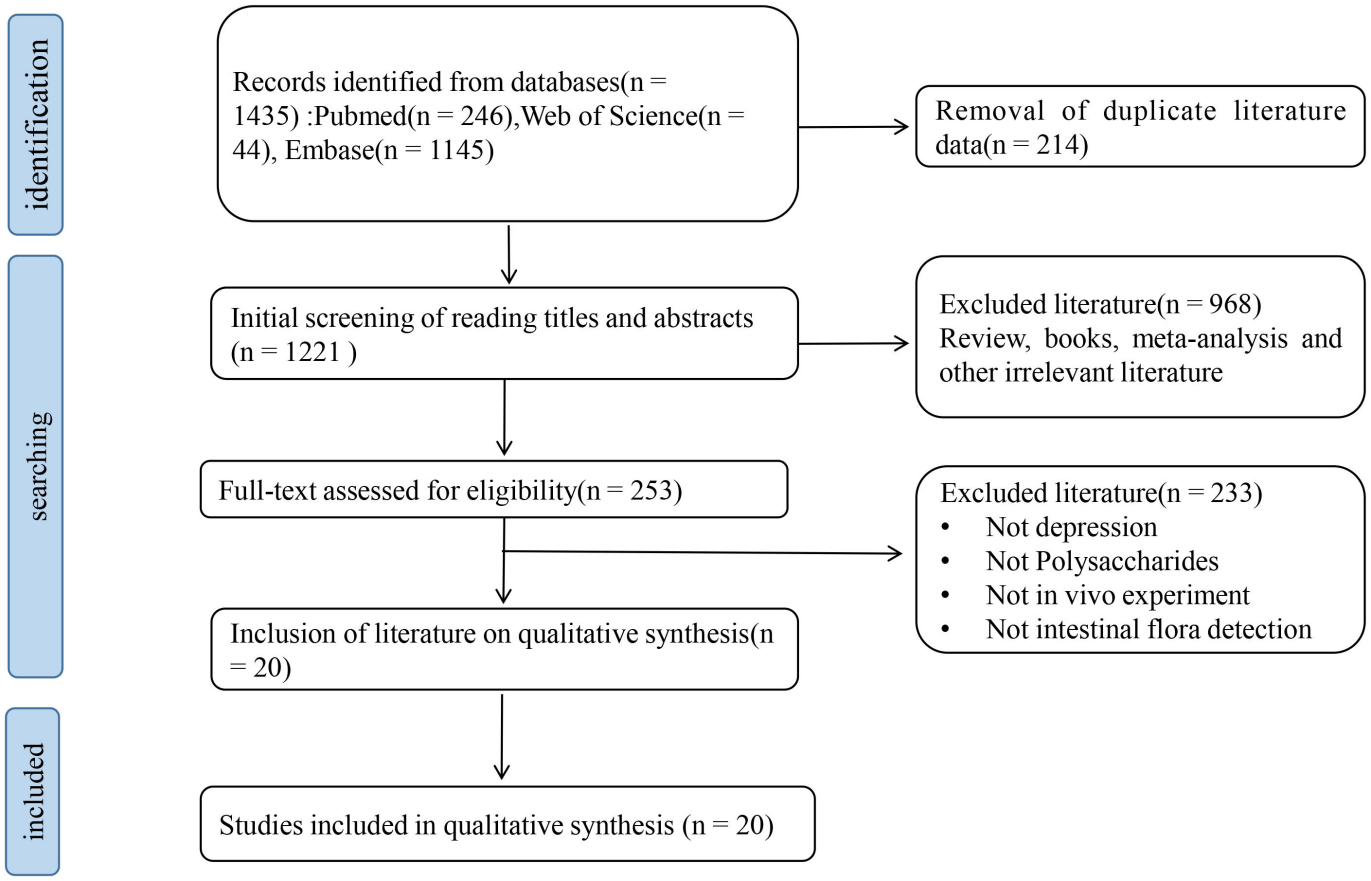
Flowchart of the literature search and screening process.

Following the predefined search strategy, a total of 1, 435 English-language records were retrieved from the three databases: 246 from PubMed, 1, 145 from Embase, and 44 from Web of Science. After importing the records into reference management software, 214 duplicate records were removed, leaving 1, 221 unique records for screening. Titles and abstracts of these 1, 221 records were screened against the eligibility criteria, and 968 records were excluded (primarily reviews, meta-analyses, conference abstracts, or studies irrelevant to the research question). The remaining 253 records underwent full-text review. Of these, 233 records were excluded for the following reasons: not an animal model of depression (n = 67), not involving polysaccharide intervention (n = 54), combination treatment with other compounds (n = 42), no gut microbiota assessment (n = 38), or full-text unavailable (n = 32). Consequently, 20 studies met all inclusion criteria. The study selection process is illustrated in the PRISMA 2020 flow diagram (**Figure 1**). The extracted data and key characteristics of the 20 included studies are summarized in **Table 1**.

**Table 1:** The origin and composition of polysaccharides.

Article	Polysaccharide	Source	Part used	Composition and ratio (or molar ratio)	Molecular weight
(Wang et al., 2024)	Polygonatum sibiricum F. Delaroche polysaccharide (PSP)	Polygonatum sibiricum F. Delaroche	Rhizomes	arabinose:glucose:glucuronic:acid:galactose:galacturonic acid:mannose:rhamnose = 13.7:82.9:3.7:36.2:4.3: 52.5:3.3:1.0	6 kDa-14 kDa
(Zhu et al., 2023)	Schisandra chinensis polysaccharide-rich (SCP)	Schisandra chinensis	Fruit	xylose:glucose:rhamnose:galactose:mannose:galacturonic acid = 0.27:5.09:0.24:1.00:0.63:2.86	681 Da, 1,148 Da, 2,628 Da, 3,232 Da
(Yang et al., 2022)	Alcohol-soluble polysaccharide (ASP)	Dendrobium officinale Kimura et Migo	Flowers	rhamnose:arabinose:fucose:mannose:glucose = 0.39:0.27:0.35:4.92:10.75	3.10 × 104 Da
(Chen et al., 2023)	Tongxieyaofang polysaccharide (TXYF-P)	Atractylodis Macrocephalae, Radix Paeoniae Alba, Pericarpium Citri Reticulatae and Radix Saposhnikoviae	NA	glucose:71.5%,protein:0.78%,polyphenol:0.6%	NA
(Zhang et al., 2024)	Cordyceps sinensis polysaccharide (CSWP-2)	Cordyceps sinensis	NA	glucose:83.03 %, mannose:7.79 %, galactose:7.61 %, glucuronic acid:0.11 %, rhamnose:0.30 %, galacturonic acid:0.47 %, xylose:0.32 %, arabinose:0.36 %	2,116.93 kDa, 58.44 kDa, 12.49 kDa, 4.00 kDa
(Yan et al., 2019)	Okra polysaccharide (OP)	okra	Fruit	mannose, rhamnose, glucuronic acid, galactosal acid, galactose, arabinose = 3.4:3.76:24.19:6.27:8.73:3.13	626 kDa
(Zhao et al., 2021)	Lycium barbarum polysaccharide (LBP)	NA	NA	NA	NA
(Chen et al., 2019)	Polysaccharide from Ginkgo biloba leaves (GPS)	Ginkgo biloba leaves	Leaves	mannose, rhamnose, glucuronic acid, galactose, arabinose = 29.73:31.54:24.81:5.72:8.30.	296.96 kDa
(Xiong et al., 2023)	Xiaoyaosan polysaccharide (PXYS)	Xiaoyaosan	NA	NA	NA
(Yu et al., 2024)	Millettia pulchra polysaccharide (YLP-1)	Millettia pulchra	Rhizomes	T-Araf, 1,3,6-Glc, 1,5-Ara, TGlcp, 1,4-Man, 1,4-Glc = 1:5: 2:6:1:25	33.0 kDa
(Wang et al., 2023)	Eucommiae cortex polysaccharides (Eps)	Eucommiae cortex	Eucommiae cortex	mannose:2.05 %, rhamnose:2.43 %, galacturonic acid:2.15 %, glucose:81.48 %, galactose:2.94 %, xylose:1.23 %, arabinose:7.10 %,fucose:0.24 %,glucuronic acid:0.39 %	6,220 Da
(Fang et al., 2023)	Corydalis yanhusuo polysaccharides (CYP)	Yanhusuo	NA	NA	NA
(Liu et al., 2023)	Cistanche deserticola polysaccharides (CDPs)	Cistanche deserticola	NA	glucose: galactose:rhamnose:fructose = 10:0.81:5.19:2.14	4.676 × 105 Da
(Zhou et al., 2023)	Paeonia lactiflora polysaccharide (PLP)	Paeonia lactiflora	Rhizome	mannose:rhamnose: glucose acid:galacturonic acid:glucose:galactose:xylose:arabinose: = 6.00:23.46:6.20:18.12:4.01:35.49:1:58.15:1.55	4.8-194.4 KDa
(Wen et al., 2025)	Gastrodia elata polysaccharide (GEP)	Gastrodia elata Blume	Rhizome	glucose:galactose = 110.11:1.00	62,480 Mw
(Luo et al., 2024)	Water-insoluble β-1,3-glucan (Wβ)	Wolfiporia cocos	Dried sclerotia	glucose(100%)	8.12 × 104 Da
(Chen et al., 2023)	Inulin	NA	NA	NA	NA
(Chen et al., 2024)	Inulin	NA	NA	NA	NA
(Joanna Szala-Rycaj et al., 2023)	Inulin (INU)	Chicory	Root	NA	NA
(Sun et al., 2024)	Ziziphus Jujube Polysaccharides (ZJP)	Air-dried jujube	Fruit	fucose:rhamnose:arabinose:galactose:glucose:xylose:mannose:fructose:galacturonic acid: glucuronic acid=0.003:0.025:0.113:0.118:0.334:0.016:0.006:0.202:0.173:0.008	83.8 kDa , 123.0 kDa

### 3.2 Quality of included studies

**Figure 2:**
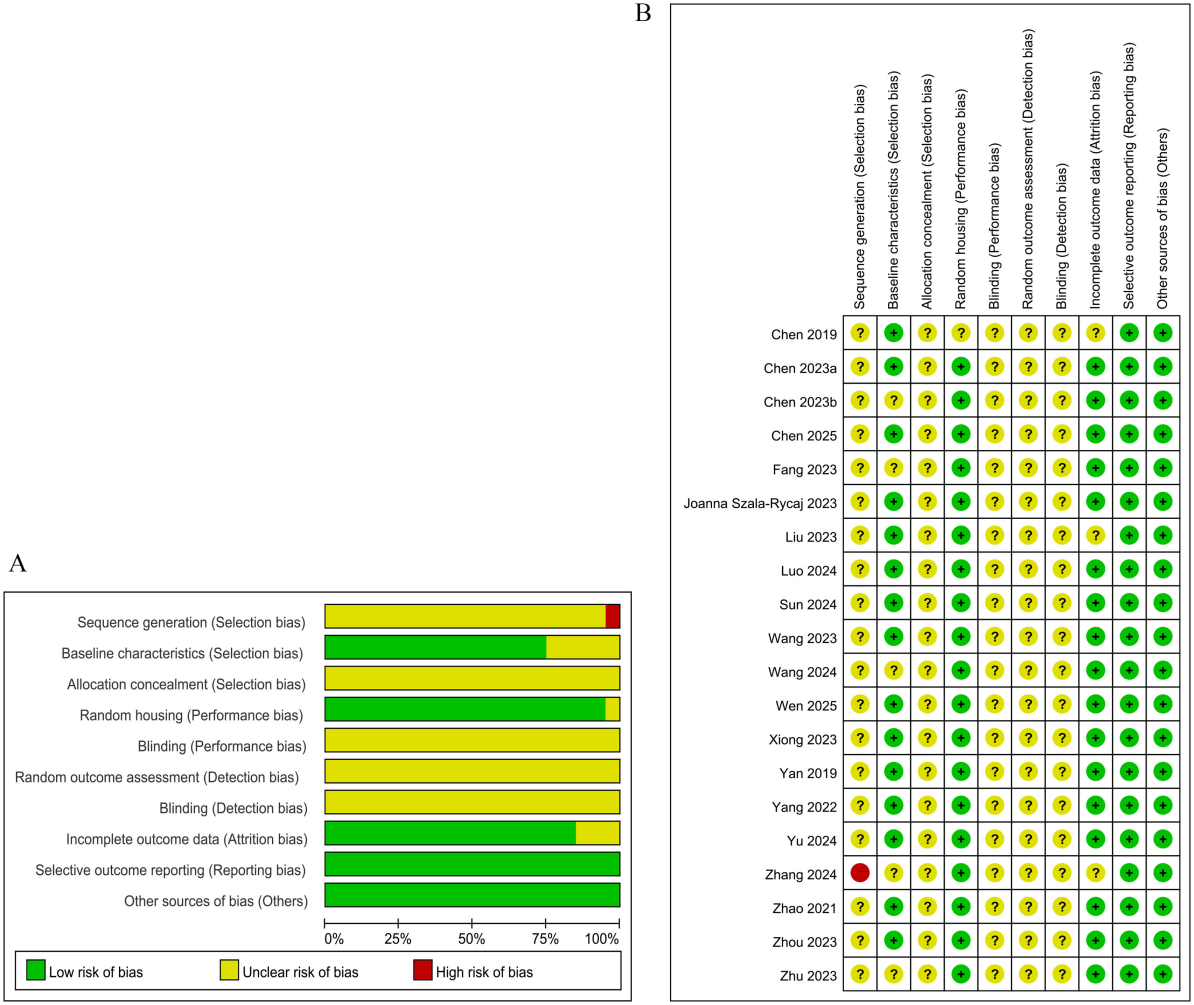
Risk of bias assessment of included animal studies.

Based on the assessment of SYRCLE’s risk of bias tool for the 20 included animal studies, the results indicated that: Regarding random sequence generation, 19 studies were rated as “unclear risk” and 1 study (36) was rated “high risk.” Regarding baseline characteristics, 15 studies were rated “low risk, ” and 5 studies (25, 26, 36–38) were classified as “unclear risk.” All included studies had a risk of uncertainty bias in areas, including allocation concealment, implementation blinding, and outcome assessment blinding. Regarding random housing, 19 studies were rated “low risk, ” and 1 study (39)was rated “unclear risk.” Regarding incomplete outcome data, 17 studies were “low risk, ” and 3 studies (36, 39, 40) were rated “unclear risk.” **Figure 2** depicts a comprehensive summary.

### 3.3 Experimental summary and general overview of polysaccharides

As summarized in **Table 1**, the 20 included studies were published between 2019 and 2025. The majority of studies (n = 16) appeared between 2023 and 2025, with a peak in 2023 (n = 9). Two studies were published in 2019, and one study each in 2021, 2022, and 2025. Three studies share the same first author (Chen Peng), while the remaining 17 were conducted by independent research groups. This systematic review included 20 studies investigating polysaccharides from 18 different sources. Among them, 16 polysaccharides were derived from a single species (16/18), whereas the remaining two were extracted from multi-herb TCM formulations (2/18). Regarding the plant parts utilized for polysaccharide extraction, eight studies did not identify the source tissue (8/20). Of the 12 studies that provided this information, the polysaccharides were extracted from the following parts: rhizomes (4/12), fruits (3/12), leaves (1/12), flowers (1/12), fungal sclerotia (1/12), bark (1/12), and root (1/12). Across the 20 included studies, glucose was the most frequently reported monosaccharide component, present in 12 studies. Among the 14 studies that provided detailed monosaccharide composition, glucose was detected in 12 (12/14).

### 3.4 Characteristics of included studies

**Table 2** illustrates that all animal models utilized in the included studies were rodents, specifically rats or mice. Mice were utilized in 14 studies, predominantly C57BL/6 mice (10/14), followed by Institute of Cancer Research (ICR) mice (2/14), Kunming (KM) mice (1/14), and Balb/c mice (1/14). Regarding body weight, 11 of the 14 mouse studies reported initial weight (11/14), which ranged from 10 g to 27 g; among these, 10 studies reported weights between 16 g and 27 g (10/11), while one study reported a lower weight of 10 ± 0.5 g (1/11). The remaining six studies utilized Sprague-Dawley (SD) rat models. All six rat studies provided data on initial body weight, which mostly ranged from 160 to 220 g. Specifically, five studies indicated a body weight between 180 and 200 g (5/6), whereas one study reported a range of 160-180 g (1/6). Animal age was reported in 8 of the 20 included studies. Among these, three studies used 8-week-old animals (3/8), three studies used animals aged 3-4 weeks (3/8), and two studies used 6-week-old animals (2/8). Sex was specified in 19 studies, and most studies (18/19) utilized male animals, whereas only 1 utilized females (1/19). The 20 studies collectively involved 997 animals, with the following strain distribution: C57BL/6 mice (593), ICR mice (82), SD rats (222), KM mice (60), and Balb/c mice (40). Regarding the construction of animal models of depression, the chronic unpredictable mild stress (CUMS) protocol dominated, and this method was adopted by 17 studies included in the analysis (17/20). The distribution of the remaining models was as follows: one study each utilized the chronic restraint stress (CRS) model (1/20), the chronic variable stress (CVS) model (1/20), and the olfactory bulbectomy (OB) mode (1/20).

**Table 2:** Basic characteristics of studies.

Article	Polysaccharide	Animal type, sex (F/M), weight (g), Age (week)	Model / model-making time(d)	Sample size	Interventions (ml/kg-1/d)	Interventions positive (mg kg-1/d)	Interventions positive (mg kg-1/d)	Interventions duration (d)
(Wang et al., 2024)	PSP	C57BL/6J mice, M, 20-23, 8	CUMS /28d	T:32; N:8	Gavage of equal amounts of water	Gavage of Fluoxetine (10)	Gavage of PSP (400)	14
(Zhu et al., 2023)	SCP	ICR mice, M, 20 ± 2 , NA	Olfactory bulbectomy	T:60; N:10	Gavage of Vehicle (20)	Gavage of Shugan jieyu capsule(200)	Gavage of SCP (800)	14
(Yang et al., 2022)	ASP	SD rats, M, 160-180, 6	CUMS /28	T:60; N:10	Gavage of aqueous solution (10)	Gavage of Fluoxetine (10)	Gavage of ASP (162)	28
(Chen et al., 2023)	TXYF-P	C57BL/6 mice, M ,20 ± 2, 3	CUS /28	T:96; N:12	Gavage of equal amounts of water	Gavage of FOS+GOS (100)	Gavage of TXYF-NP (200)	28
(Zhang et al., 2024)	CSWP-2	Kunming mice, M, NA, 4	CRS /14	T:60; N:10	Gavage of equal amounts of water	Gavage of sertraline (7.6)	Gavage of CSWP-2-L (200)	14
(Yan et al., 2019)	OP	C57BL/6 mice, M, 18-22, NA	CUMS /28	T:90; N:10	NA	NA	Gavage of OP (400)	14
(Zhao et al., 2021)	LBP	SD rats, F, 200 ± 20, NA	CUMS /21	T:24; N:8	Gavage of equal amounts of water	NA	Gavage of LBP (40)	14
(Chen et al., 2019)	GPS	Balb/c mice, M, NA, 3-4	CUMS /30	T:40; N:10	Gavage of equal amounts of PBS	Gavage of paroxetine (30)	Gavage of GPS (300)	28
(Xiong et al., 2023)	PXYS	SD rats , M, 180 ± 20, 8	CUMS /42	T:36; N:6	Gavage of equal amounts of water	Gavage of fluoxetine (2)	Gavage of PXYS (1200)	42
(Yu et al., 2024)	YLP-1	SD rats, M, 200 ± 20, NA	CVS /35	T:30; N:5	NA	Gavage of fluoxetine (3)	Gavage of YLP-1 (600)	35
(Wang et al., 2023)	Eps	ICR mice, M, 17-27, NA	CUMS /28	T:22; N:11	NA	NA	Gavage of Eps (400)	14
(Fang et al., 2023)	CYP	C57BL/6 mice, M, 20 ± 2, NA	CUMS /56	T:32; N:8	Gavage of equal amounts of water	Gavage of fluoxetine (2)	Gavage of CYP (200)	28
(Liu et al., 2023)	CDPs	SD rats, M, 180-220, 6	CUMS /28	T:24; N:6	NA	Gavage of Paroxetine (5)	Gavage of CDPs (200)	28
(Zhou et al., 2023)	PLP	C57BL/6 mice, M, 18 ± 2, NA	CUMS /56	T:60; N:10	Gavage of equal amounts of water	Gavage of fluoxetine (8)	Gavage of PLP (200)	28
(Wen et al., 2025)	GEP	C57BL/6 mice, M, 20 ± 2, 8	CUMS /28	T:72; N:12	Gavage of equal amounts of water	Gavage of fluoxetine (2.6);	Gavage of GEP (200)	28
(Luo et al., 2024)	Wβ	SD rats, M, 200 ± 20, NA	CUMS /32	T:48; N:8	NA	Gavage of fluoxetine (8)	Gavage of Wβ (500)	35
(Chen et al., 2023)	inulin	C57BL/6 mice, NA, NA, NA	CUMS /84	T:80; N:10	NA	NA	NA	84
(Chen et al., 2024)	inulin	C57BL/6 mice, M, 20 ± 1, NA	CUMS /56	T:60; N:10	NA	NA	inulin(4000)	56
(Joanna Szala-Rycaj et al., 2023)	INU	C57BL/6 mice, M, 20-25, 6	CUMS /42	T:35; N:7	Injection of equal amounts of water	Gavage of fluoxetine (12)	Gavage of INU (66)	70
(Sun et al., 2024)	ZJB	C57BL/6 mice, M, 10 ± 0.5, NA	CUMS /44	T:36; N:6	Gavage of equal amounts of water	NA	Gavage of ZJB (800)	30

Regarding interventions within model control groups, seven studies failed to specify the control treatment. Among the 13 studies that provided details, nine used clean drinking water or normal water as the control, one employed phosphate-buffered saline, and three used other vehicles (an aqueous solution, an unspecified vehicle, or intraperitoneal water injection). Regarding positive control interventions, fluoxetine was the most frequently utilized agent (9/20 studies), with doses ranging from 2 to 12 mg·kg^-1^ (specifically: 10 mg·kg^-1^ in two studies, 2 mg·kg^-1^ in two studies, 8 mg·kg^-1^ in two studies, and single studies using 12, 3, and 2.6 mg·kg^-1^). Other positive controls included paroxetine (two studies: 5 and 30 mg·kg^-1^), sertraline (one study: 7.6 mg·kg^-1^), Shugan Jieyu Capsule (one study: 200 mg·kg^-1^), FOS+GOS (one study: 100 mg·kg^-1^), and St. John’s wort (one study: 150 mg·kg^-1^). Additionally, six studies did not report or implement any positive drug intervention. The therapeutic interventions included 18 distinct polysaccharides, with administered doses ranging from 40 to 4000 mg·kg^-1^. Except for two studies where the administration route was clearly undefined, all other interventions were implemented by oral gavage (18/20). The treatment duration of polysaccharide interventions was reported in all 20 included studies. The administration periods were as follows: 14 days (6/20), 28 days (7/20), 30 days (1/20), 35 days (2/20), 42 days (1/20), 56 days (1/20), 70 days (1/20), and 84 days (1/20).

**Figure 3:**
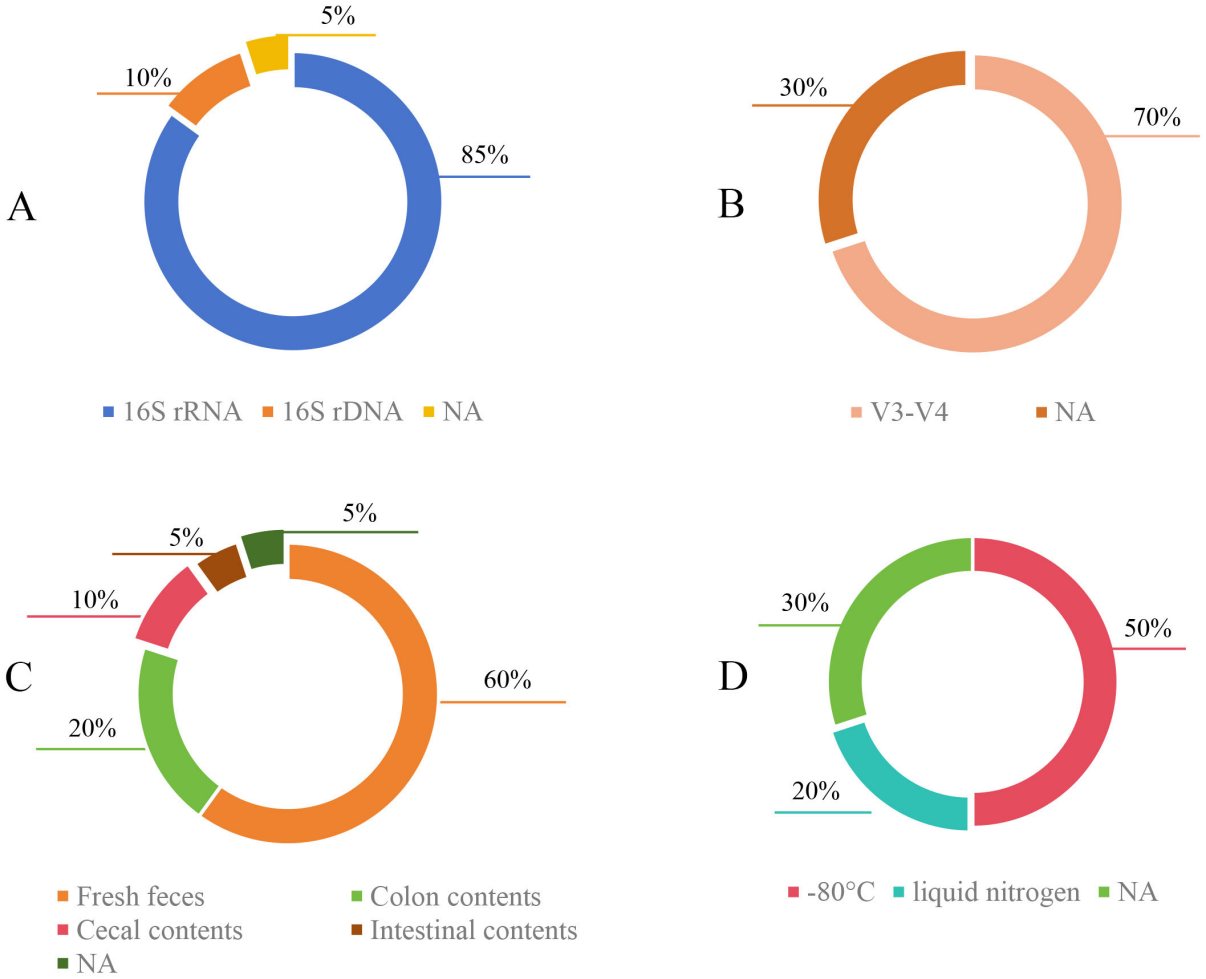
Methodological characteristics of gut microbiota analysis in the included studies. **(A)** Distribution of sequencing approaches; **(B)** Distribution of targeted hypervariable regions; **(C)** Distribution of microbial sample sources; **(D)** Distribution of sample preservation methods.

**Figure 3** indicates that in gut microbiota research methodologies, 16S rRNA sequencing was the most widely utilized analytical approach (17/20, 85%), whereas two studies employed 16S rDNA-based detection (2/20, 10%), and one study did not specify the methodological details (1/20, 5%). Of the 20 included studies, V3-V4 hypervariable regions were the primary amplification targets in 14 investigations (14/20, 70%), and the remaining studies failed to report specific sequenced regions (6/20, 30%). Regarding sample types for microbial analysis, fecal specimens were utilized most often (12/20, 60%), followed by colonic content (4/20, 20%) and cecal content (2/20, 10%). Studies that were generically referred to as “intestinal content” and those that did not specify sample type each accounted for 5% (1/20, respectively). For sample preservation conditions, most studies stored specimens at -80 °C (10/20, 50%), whereas 20% (4/20) employed liquid nitrogen storage, and 30% (6/20) did not report storage parameters.

### 3.5 Behavioral improvement indicators

**Table 3** indicates that body weight was reported in 13 of the 20 included studies. These studies mostly reported a decrease in animal body weight following the induction of depression models. Administration of various polysaccharides was associated with reversal of this trend, leading to a quantifiable increase in body weight (13/13). These findings suggest that polysaccharides may have therapeutic potential in alleviating depression-associated weight loss. Immobility duration in the forced swim test (FST, 15/15) and tail suspension test (TST, 12/12) was decreased in all reported instances, providing preliminary evidence for the amelioration of behavioral despair. The sucrose preference test (SPT, 12/13) indicated improvement in 12 studies, suggesting mitigation of anhedonia, a key feature of depression. Moreover, open field test (OFT, 15/15) activity was consistently enhanced, suggesting that the effects were unlikely to be due solely to locomotor stimulation and may reflect improved exploratory behavior. Results from the elevated plus maze (EPM, 6/6), light-dark test (LDT, 2/2), and Morris water maze (MWM, 2/2) mostly indicated anxiolytic effects alongside the primary antidepressant efficacy.

**Table 3:** Regulation of intestinal flora by polysaccharides and their improvement effect on depression.

Article	Polysaccharide	Weight and behavior	Inflammatory levels	Oxidative stress	Biochemical indicators of depression	Intestinal barrier	NMDS	SCFAs	F/ B
(Wang et al., 2024)	PSP	Weight↑,TST↓,FST↓,OFT↑	LPS↓	NA	5-HT↑,CORT↓	ZO-1↑,Occludin↑,Claudin1↑;	Chao1↑,Simpson↑;PCoA: convergent to the control	NA	↓
(Zhu et al., 2023)	SCP	Weight↑,SPT↑,FST↓,TST↓,OFT↑	NA	T-SOD↑,MDA↓, CAT↑,H2O2↓	Brain:BDNF↑,PSD95↑;Serum ACTH↓,CORT↓	NA	Chao1↑,ACE↑,Sobs indices↑;PCoA: closely clustered to the controls	NA	NA
(Yang et al., 2022)	ASP	NA,SPT↑,OFT↑,FST↓	IL-10↑,TNF-a↓,IL-1β↓	NA	5-HT↑,BDNF↑	ZO-1↑	Chao1↑,Shannon↑,Simpson↑;PCoA: similarities to the controls	↑(acetate,hexanoic,butyrate)	NA
(Chen et al., 2023)	TXYF-P	Weight↑,SPT↑,FST↓,TST↓	NA	NA	Brain:ACTH↓,CORT↓;hypothalamus:Crhr1↓,Nr3c1↓,Nr3c2↓;Hippocampus:Crhr1↑,Nr3c1↑,Nr3c2↑;(↑Gria1,Grin1, Grm1,Grm4,Gabra1,Gabra2,Gabbr1,Gabbr2)Gria2↓,5-HT↑,5-HIAA↓	NA	Chao1↑,Shannon↑,Simpson↑,ACE↑,observed-species↑;PCoA: microbiota structure was different	NA	↓
(Zhang et al., 2024)	CSWP-2	Weight↑,OFT↑,FST↓,TST↓	TNF-α↓,IL-1β↓,IL-10↑,NF-κB↑,IL-2↑,IL- 6↑	NA	5-HT↑,NE↑,BDNF↑,CORT↓,CRH↓,ACTH↑	NA	Chao1↓,ACE↓,Goods coverage↑,observed species↓,richness↓;PCoA: separation among the groups	NA	NA
(Yan et al., 2019)	OP	NA,OFT↑,EPM↑,FST↓,TST↓	TNF-α↓, IL-1β↓, IL-6↓	NA	NA	NA	Chao1↑,Shannon↑,Simpson↓;	isovaleric↓,acetic↑,propionic↑,butyric↑	↑
(Zhao et al., 2021)	LBP	Weight↑,SPT↑,OFT↑,TST↓	NA	NA	CORT↓,GABA↓,5-HT↓,CCK↓	NA	ACE↑,Sobs indices↑,Chao1↑,Shannon↑,Simpson↓	NA	↑
(Chen et al., 2019)	GPS	Weight↑,OFT↑,FST↓,TST↓	NA	NA	5-HT↑,DA↑	NA	Shannon↑,Simpson↓;NMDS& PCoA: similar structure to that of the control	NA	NA
(Xiong et al., 2023)	PXYS	Weight↑,SPT↑,OFT↑	NA	NA	NA	NA	ACE↓,Chao↓,Shannon↓;NMDS& PCoA: difference of the control.	NA	↑
(Yu et al., 2024)	YLP-1	Weight↑,SPT↑,FST↓,TST↓	IL-1β↓,IL-18↓	NA	5-HT↑,DA↑,CORT↓	NA	NA;PCoA: disturbed gut microbiota community could be regulated	↑(glacial,acetic,butyric,valeric,isovaleric,hexanoic)	NA
(Wang et al., 2023)	Eps	NA,FST↓,TST↓,EPM↑,OFT↑	TNF-α↓,MCP-1↑	NA	5-HT↑,5-HIAA↑	ZO-1↓	Chao↑;Bray-Curtis&PCoA: closer to that of the Control	NA	NA
(Fang et al., 2023)	CYP	NA,SPT↑,TST↓,FST↓	NA	NA	5-HT↑,DA↑,NE↑,BDNF↑,TPH-2↑	NA	Chao↑, Shannon↑;NMDS& PCoA: clear separation of microbiota in the groups	↑(acetic,propionic,butyric,isobutyric,valeric,isovaleric,caproic)	↓
(Liu et al., 2023)	CDPs	Weight↑,SPT↑,FST↓,OFT↑,MWM↑	IL-10↑,TGF-β↑,IL-17↑,IL-22↓	NA	NA	NA	Chao↓,Shannon↓,Simpson↓;NMDS& PCoA: significantly altered of gut microbiome of rats induced by CUMS	↑(acetic,butyric,lactic)	↑
(Zhou et al., 2023)	PLP	Weight↑,SPT↑,FST↓,OFT↑	IL-1β↓, IL-6↓,TNF-α↓	NA	5-HT↑	NA	Chao↓,Shannon↑;PCoA: similar of CON	NA	↓
(Wen et al., 2025)	GEP	NA,SPT↓,FST↓	NA	SOD↑,MDA↓,Keap1↓,c-Nrf2↓,n-Nrf2↑	BDNF↑	NA	Observed species↑;Shannon↑;PCoA: GEP groups were more similar to the control	NA	↓
(Luo et al., 2024)	Wβ	NA,SPT↑,OFT↑,TST↓,FST↓	LPS↓,IL-1β↓,IL-6↓,TNF-α↓,IL-18↓	NA	5-HT↑,DA↑,Glu↓	NA	NA;PCoA:was clearly separated from the normal rats	↑(acetic,propionic,butyric,isobutyric,valeric,isovaleric,hexanoic)	↓
(Chen et al., 2023)	inulin	NA,SPT↑,OFT↑,LDT↑,EPM↑	IL-1β↓,IL-6↓,TNF-α↓	NA	5-HT↑,NA↑,BDNF↑	NA	Chao↓,Shannon↓,Simpson↓;PCoA:significantly affected the microbe	↑(acetic,propioni,butyric)	NA
(Chen et al., 2024)	inulin	Weight↑,SPT↑,OFT↑,LDT↑,EPM↑	LPS↓,IL-1β↓,IL-6↓,TNF-α↓,NLRP3↓,Caspase1↓	NA	NA	ZO-1↑	NA;differed significantly with CUMS group	NA	NA
(Joanna Szala-Rycaj et al., 2023)	INU	Weight↑,EPM↑,FST↓,MWM↓	NA	NA	NA	NA	Chao↓,Shannon↑;PCoA:high similarity between the studied profiles grouped	NA	↓
(Sun et al., 2024)	ZJB	Weight↑,TST↓,OFT↑,EPM↑	TNF-α↓,IL-1β↓,IL-6↓,IL-2↓	NA	5-HT↑,DA↑	NA	Chao↓,Simpson↓;PCA: verlapped with each control	↓(acetic,propionic butyric)	↓

### 3.6 Inflammatory and oxidative stress indicators

The analysis of inflammation and oxidative stress markers suggested that natural polysaccharide interventions were associated with ameliorated inflammatory conditions and oxidative stress in depression models. The administration of natural polysaccharides was associated with inhibition of pro-inflammatory factors, with tumor necrosis factor-alpha (TNF-α, 9/9) and interleukin-1beta (IL-1β, 9/9) reduced in all studies, whereas interleukin-6 (IL-6, 6/7) exhibited a decrease. Simultaneously, the anti-inflammatory cytokine interleukin-10 (IL-10, 3/3) was elevated, signifying a transition toward anti-inflammatory homeostasis. TGF-β rose in one study, whereas IL-17 increased in one study and IL-22 decreased in one study. At the molecular mechanism level, NLRP3 and Caspase-1 exhibited a decrease in one study, whereas their downstream factor IL-18 exhibited a decline in two studies. LPS exhibited a decrease (3/3) in all three studies.

A systematic analysis of 20 studies suggested that natural polysaccharides may be associated with improvements in oxidative stress markers in animal models of depression. Total superoxide dismutase (T-SOD/SOD) exhibited an increase in activity in both studies (2/2). Catalase (CAT) exhibited an increase in activity in one study (1/1). Malondialdehyde (MDA) exhibited a decrease in concentration in both studies (2/2), whereas hydrogen peroxide (H_2_O_2_) decreased in one study (1/1). At the molecular mechanism level, Keap1 was downregulated in one study (1/1), and it was observed that the cytoplasmic Nrf2 (c-Nrf2) level decreased (1/1), whereas the level of nuclear Nrf2 (n-Nrf2) increased (1/1).

### 3.7 Neurobiochemical indicators

Analysis of physiological and biochemical parameters from the included studies indicated that natural polysaccharide interventions mostly modified essential neurobiological markers in depression models. Serotonin 5-hydroxytryptamine (5-HT) was increased in 13 of the 14 studies that reported this measure with one study reporting a decrease. Dopamine (DA) and norepinephrine (NE) exhibited increases in 5/5 and 3/3 studies investigating these neurotransmitters, respectively. Assessment of neuroendocrine function indicated that corticosterone (CORT) was diminished in 6/6 studies, whereas adrenocorticotropic hormone (ACTH) decreased in 2/3 relevant studies. CRH exhibited a decrease in one study, indicating its regulatory ability over the on-axis source of HPA. Notably, brain-derived neurotrophic factor (BDNF) was upregulated in 6/6 studies that evaluated this marker. Moreover, postsynaptic density protein 95 (PSD95) increased in one study, indicating its role in facilitating synaptic maturation. Multiple receptor subunits of glutamate receptors (Gria1, Grin1, Grm1, and Grm4) and GABA receptors(Gabra1, Gabra2, Gabbr1, and Gabbr2) were increased in another study. Other studies have indicated that tryptophan hydroxylase-2 (TPH-2) increased in one study, indicating that it may function by enhancing 5-HT biosynthesis.

### 3.8 Intestinal barrier

Assessment of intestinal barrier markers suggested that natural polysaccharide intervention mostly enhanced gut barrier integrity in depression models. Zonula occludens-1 (ZO-1, 3/4) expression was upregulated in most relevant studies. Moreover, increased expression of occludin and claudin-1 was reported in the individual studies that assessed each protein. Despite one study reporting lower ZO-1 levels, the majority of evidence suggests that natural polysaccharides may enhance the expression of essential tight junction proteins. These findings suggest that preserving intestinal barrier integrity and strengthening the mucosal barrier may constitute a mechanism underlying the antidepressant effects of natural polysaccharides.

### 3.9 Characteristics and metabolic functions of the gut microbiota

The assessment of gut microbiota diversity parameters suggested that administration of natural polysaccharides was mostly associated with improved microbial community structure in depression models. Analysis of the 20 included studies showed that the Chao1 index (15/20) and the Shannon index (12/20) were the principal metrics used to assess α-diversity. For the Chao1 index, most studies (8/15) reported an increase, which may imply enhanced species richness, while 7 studies (7/15) reported a decrease, indicating heterogeneity in the microbiota response. The Shannon index showed an upward trend in 9 of the 12 studies that reported this metric (9/12), with three studies showing a decline (3/12); this has been interpreted as a possible increase in species diversity and ecological stability. The Simpson index was cited as a measure of community dominance in nine studies (9/20); among these, six studies reported a decrease (6/9), suggesting a reduction in single-species dominance and a trend toward more uniform species distribution. The ACE index (5/20) and the observed species index (5/20), used as auxiliary measures of species richness, showed an upward trend in most studies (ACE: 3/5; observed species: 4/5), which may reflect a positive influence of polysaccharides on microbiota richness. A systematic analysis of the 20 included studies indicated that principal coordinates analysis (PCoA) was the most commonly used method for evaluating β-diversity (16/20). Among these, four studies combined PCoA with non-metric multidimensional scaling (NMDS, 4/20), one study used Bray-Curtis distance (1/20), and one study used principal component analysis (PCA, 1/20). Overall, polysaccharide intervention was associated with a reversal of gut microbiota dysbiosis induced by depression models and with a shift toward a composition more similar to that of healthy control groups.

A systematic analysis of the Firmicutes to Bacteroidetes (F/B) ratio suggested that natural polysaccharides may be associated with modulation of intestinal microbiota composition in depression models. Among the 12 studies that reported this parameter, 8 reported a reduction in the F/B ratio (8/12), whereas the remaining 4 studies (4/12) reported increased values. This predominant pattern of reduction suggests that natural polysaccharides may counteract depression-associated increases in the F/B ratio, potentially contributing to restoration of microbial equilibrium. Although inconsistencies were observed in a few reports, the overall evidence indicates that natural polysaccharides may help reconfigure gut microbial community architecture in a direction generally considered beneficial.

The assessment of short-chain fatty acid (SCFA) profiles suggested that administration of natural polysaccharides was associated with changes in microbial metabolic activity in the intestinal environment of depression models. Of the 20 included studies, 8 provided data on SCFAs. Most of these studies (6/8) reported a general increase in SCFA levels after polysaccharide intervention, whereas one study (1/8) reported an overall decrease, and another study(1/8) did not specify the overall directional change. This trend suggests that enhancing the production of beneficial microbial metabolites may be a potential pathway through which polysaccharides exert their effects. Of the 20 included studies, changes in eight SCFAs and organic acids were reported. Analysis of these studies showed increases in acetic acid (6/7), butyric acid (6/7), propionic acid (4/5), hexanoic acid (4/4), valeric acid (3/3), and isobutyric acid(2/2). In addition, isovaleric acid showed an upward trend (3/4), and lactic acid production increased in the one study that reported it (1/1). These results suggest that natural polysaccharide interventions may be associated with enhanced synthesis of key SCFAs, particularly acetic, butyric, and propionic acids.

**Figure 4:**
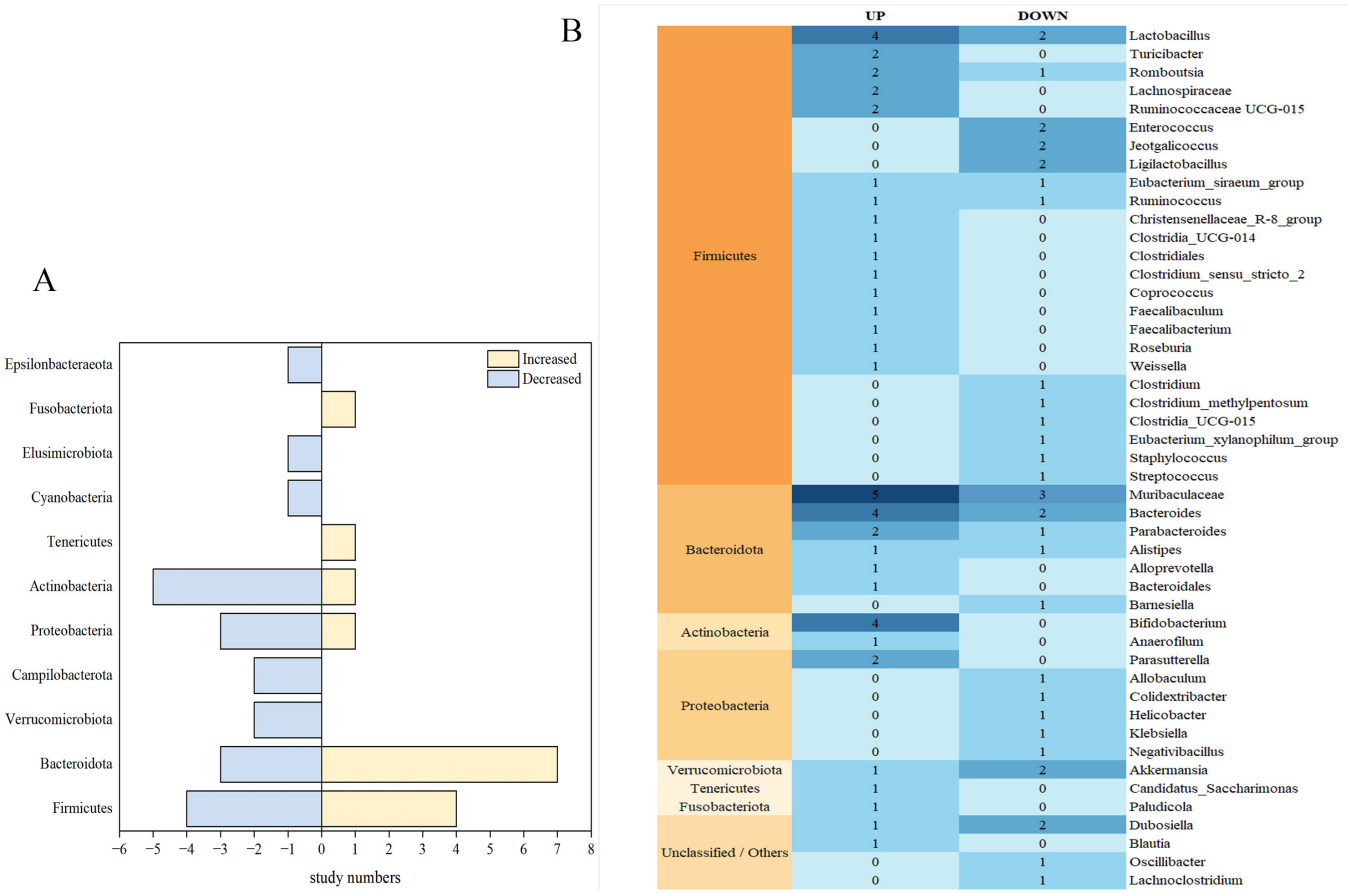
Research diagram of the phylum and genus of bacteria. **(A)** Number of studies on the increase or decrease of bacterial phylum abundance after polysaccharide treatment; **(B)** Number of studies in which bacterial phyla and genus abundance increased or decreased after polysaccharide treatment.

As shown in **Figure 4**, analysis of the 20 included studies suggested structural reorganization among 11 bacterial phyla. Bacteroidota showed an increase in 7/10 studies and a decrease in 3/10, which may support its role as a primary polysaccharide-degrading phylum. Firmicutes exhibited a balanced variation, with an increase in 4/8 studies and a decrease in 4/8, possibly reflecting functional diversification among its constituent genera. Actinobacteria decreased in 5/6 studies and increased in 1/6, which might indicate preferential regulation of alternative metabolic pathways. Proteobacteria decreased in 3/4 studies and increased in 1/4, whereas Verrucomicrobiota (2/2) and Campylobacterota (2/2) showed a decrease in all reported instances, which has been interpreted as potentially beneficial modulation of the gut microenvironment. Minor phyla showed directional changes: Tenericutes (1/1) and Fusobacteriota (1/1) increased, whereas Cyanobacteria (1/1), Elusimicrobiota (1/1), and Epsilonbacteraeota(1/1) decreased.

At the genus level, analysis of 48 genera suggested that Bifidobacterium increased in 4/4 studies; Lactobacillus increased in 4/6 studies and decreased in 2/6; Bacteroides increased in 4/6 studies and decreased in 2/6; Muribaculaceae increased in 5/8 studies and decreased in 3/8. SCFA-producing genera, including Roseburia (1/1), Turicibacter (2/2), and Parasutterella (2/2), showed an increase in all reported instances. Potentially pathogenic genera tended to decrease: Akkermansia decreased in 2/3 studies, whereas Enterococcus (2/2), Ligilactobacillus (2/2), and Jeotgalicoccus (2/2) showed a decrease in all studies that reported them. Variable responses were observed for Dubosiella (increased in 1/3, decreased in 2/3), Alistipes (increased in 1/1, decreased in 1/1), and the *Eubacterium siraeum* group (increased in 1/1, decreased in 1/1). The remaining 26 genera, each reported in single studies, showed a balanced distribution (13/26 increased, 13/26 decreased).

## 4 Discussion

**Figure 5:**
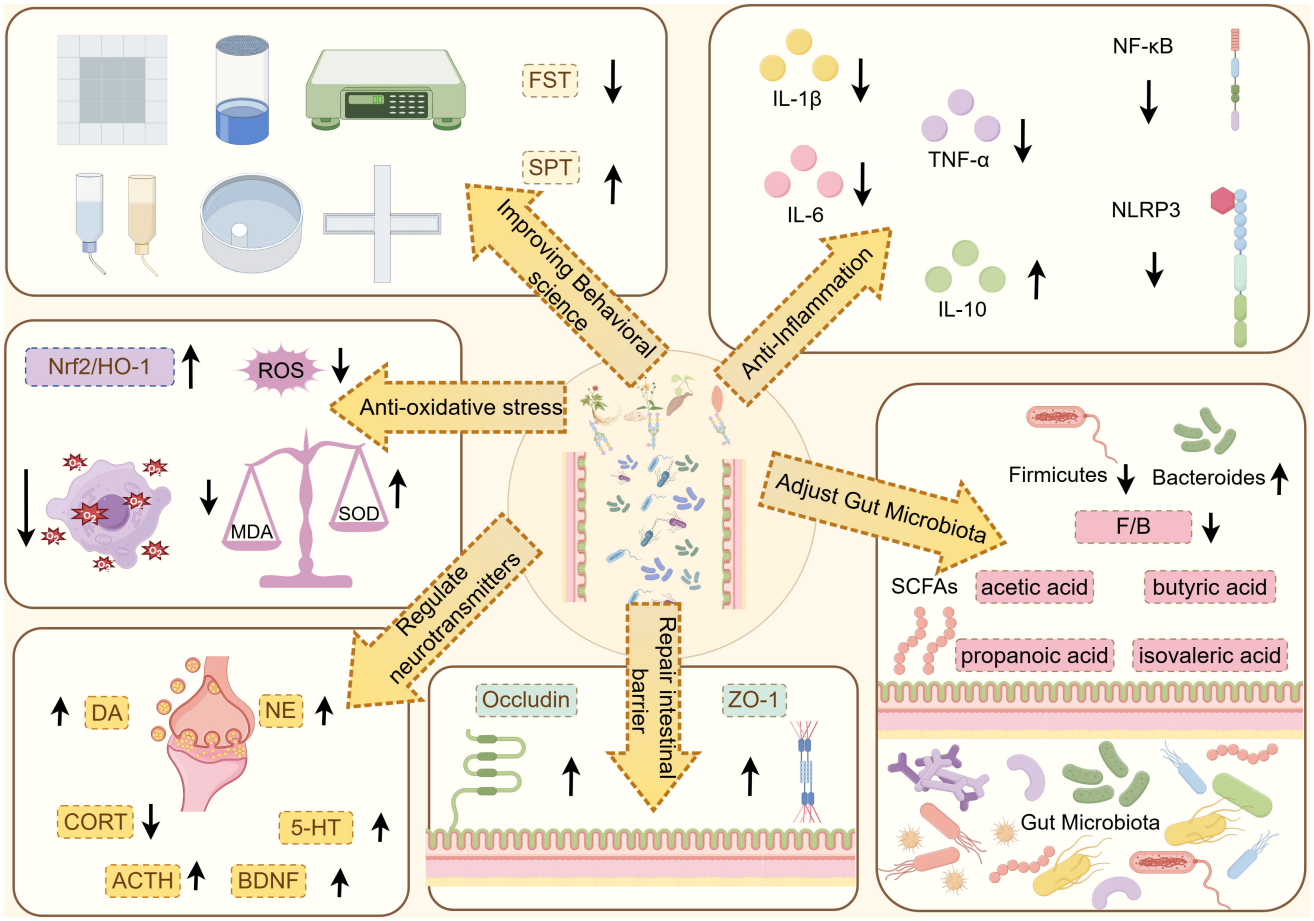
Proposed mechanisms underlying the antidepressant-like effects of polysaccharides in animal models. Polysaccharide intervention may influence multiple pathways, including inflammation, oxidative stress, neurotransmitter and HPA axis regulation, intestinal barrier integrity, gut microbiota composition, and SCFA production.

This systematic review synthesized data from 20 preclinical studies that evaluated the antidepressant effects of natural polysaccharides in animal models of depression. The outcomes assessed included behavioral and physiological indicators, neurochemical parameters, markers of neuroinflammation and oxidative stress, gut barrier integrity, gut microbial community structure, and microbial metabolites. Collectively, these studies suggested the antidepressant efficacy of natural polysaccharides and pointed to underlying mechanisms that may involve neurotransmitter modulation, regulation of oxidative stress and inflammatory responses, and pathways related to the gut-brain axis, as illustrated in **Figure 5**. It is important to note that the 20 included animal studies had a high risk of bias (most domains rated as ‘unclear’ according to SYRCLE), and the evidence is largely correlational. Therefore, the following mechanistic interpretations should be viewed as hypothesis generating rather than conclusive.

### 4.1 Behavioral and neurobiological improvements

A previous study (41)reported that body weight changes in animal models of depression may serve as an indicator for assessing depressive-like behaviors and the efficacy of interventions. This finding aligns with the “poor appetite and physical weakness” symptoms observed in human patients with depression (42, 43). Reversing the weight loss associated with the depression model may enhance the core physiological condition of depression (44). Findings from 11 studies suggested that natural polysaccharides may be associated with reversal of weight loss in depression models. This phenomenon holds physiological importance, as weight maintenance involves a complex balance of energy consumption, metabolic efficacy, and neuroendocrine regulation (45, 46). Previous studies have indicated that weight loss in patients with depression is closely associated with the overactivity of the HPA axis (47, 48). The continuous increase in corticosterone levels may contribute to appetite suppression and energy metabolism disorders through the glucocorticoid receptor signaling pathway (49). Natural polysaccharides were reported to improve feeding behavior through modulation of appetite-related neuropeptide expression. This modulation involves the upregulation of orexigenic neuropeptides and the downregulation of anorexigenic neuropeptides (50). Moreover, the regulatory effect of polysaccharides on the gut microbiota may indirectly influence body weight by increasing SCFA production, improving metabolic efficiency, and enhancing nutrient absorption (51).

Behavioral assessments are commonly used criteria for evaluating the validity of animal models of depression and the efficacy of interventions (52). SPT is an important indicator for assessing the core symptom of depression, “anhedonia”, whereas FST and TST are commonly used behavioral studies for assessing “behavioral despair” (53, 54). OFT and EPM reflect animals’ inclination to explore and their adventurous nature in new environments (55). These behavioral assessments illustrate how natural polysaccharides may be associated with changes in depression-like behaviors, providing preliminary evidence of their potential antidepressant efficacy. A summary of the 20 preclinical studies indicated a decrease in immobility duration in FST and TST, suggesting that polysaccharide treatment generally ameliorated “behavioral hopelessness” in depressed animals. Of the 13 studies mentioning SPT, 12 observed that depressed animals exhibited an increased preference for sugar water following polysaccharide treatment, suggesting that such treatment generally reversed the “anhedonia” associated with depressed animals. Moreover, the OFT, EPM, and LDT were all reported to reflect the animals inclination to explore their adventurous nature in new environments. The study indicated that polysaccharide treatment positively influenced all these indicators (56).

The reduction in immobility duration in the FST and the TST is the standard evidence that natural polysaccharides exert a central antidepressant effect (53). The alleviation of this “desperate” state of behavior has conventionally been associated with the upregulation of the monoamine neurotransmitter system in the brain (57). These results point to a dual mechanism: the active components or metabolites of natural polysaccharides may either penetrate the blood-brain barrier, or indirectly regulate neurochemical signaling along the gut-brain axis. Both pathways are critical for emotional and behavioral activation. The reversal of anhedonia noted in the SPT is more crucial. Anhedonia is the core negative symptom of depression, primarily associated with the dysfunction of the midbrain limbic dopamine system (58). Polysaccharide treatment was reported to reinstate animals’ sensitivity to sucrose rewards, suggesting involvement of the dopaminergic reward pathway (59). Some studies also indicated improvement in Morris water maze (MWM) performance, suggesting that the antidepressant-like effects may extend to hippocampal-dependent spatial learning and memory, possibly indicating protection of hippocampal neurons and promotion of neural plasticity (40). A study observed positive effects of intervention measures on the progeny of depressed female mice. This suggests that natural polysaccharides may induce a positive developmental programming effect on the offspring’s brain through modulation of the maternal internal environment during pregnancy and delivery. In this way, they may partially prevent the passage of depression-like phenotypes from mother to offspring (60).

### 4.2 Neurochemistry and endocrinology

Numerous studies have suggested elevated levels of 5-HT, DA, and NE in brain tissue and serum, providing correlational evidence of the central nervous system regulatory activity of natural polysaccharides. The upregulation of the 5-HT system aligns with the mechanism of traditional SSRI drugs and is associated with enhancement in mood, appetite, and sleep (61). The synergistic increase of DA and NE offers a more comprehensive understanding of the neurochemical basis of the reversal of anhedonia and the enhancement of exploratory motivation observed in behavioral science (62). TPH-2 upregulation was also noted. TPH-2 is the rate-limiting enzyme for serotonin (5−HT) synthesis in the brain (63). Increased expression of this enzyme suggests that natural polysaccharides may act through multiple mechanisms: regulating neurotransmitter release or reuptake, and promoting *de novo* 5-HT biosynthesis at the gene transcription level. This may offer a broader strategy for correcting monoamine deficiency in depressive states. The observed increase in 5-HT in colonic tissue suggests that polysaccharides may indirectly affect the availability of peripheral 5-HT precursors to the central nervous system by regulating the metabolism of intestinal chromaffin cells, potentially providing a potential molecular link underlying communication within the “gut-brain axis” (64).

Hyperactivation of the HPA axis constitutes a neuroendocrine disorder central to depression (65). The decrease of CRH, ACTH, and CORT levels in the study suggests that the hyperactive state of the HPA axis may have been reduced (36). In the hypothalamus, the downregulation of pro-stress CRH receptor 1 (Crhr1) and glucocorticoid receptors (Nr3c1, Nr3c2) may contributes to a reduction of the “regulatory point” of the stress response (66). In the hippocampus, the upregulation of the receptor expression enhances the inhibitory feedback of glucocorticoids on the HPA axis, suggesting a possible restoration of HPA axis function toward baseline levels (67). The overall increase in BDNF may be considered as preliminary evidence that natural polysaccharides may exert neuroprotective and restorative benefits. BDNF supports neuronal survival and essentially plays a critical role in synaptic formation and maturation (68). This is suggested by the upregulation of postsynaptic PSD95, a core scaffold protein essential for excitatory synaptic maturation and function (69). The increase signifies the fortification of synaptic architecture and the improvement of signal transmission efficiency (70). Furthermore, the general upregulation of glutamic acid and GABA receptor subunits, together with a reduction in basal glutamate levels, may enhances synaptic strength and signal processing capacity. At the same time, it may mitigate potential excitotoxicity, potentially promoting a healthier excitation/inhibition balance at a higher functional level (71). Natural polysaccharides may enhance neurogenesis, synaptic plasticity, and the restoration of monoaminergic neural transmission, which may be reflected at the behavioral level as amelioration of depressive-like behaviors.

### 4.3 Immunomodulation

Previous studies have shown that natural polysaccharides may alleviate neuroinflammation and oxidative damage associated with depression. They achieve this by regulating inflammatory signaling pathways and oxidative stress responses through multiple targets. This provides a key immunological mechanism underlying their antidepressant effects (72, 73). Regarding inflammatory regulation, natural polysaccharides indicate pleiotropic immunomodulatory properties (74, 75). All studies observed the persistent reduction of pro-inflammatory factors TNF-α and IL-1β, a finding of pathophysiological significance. TNF-α, as a key initiating factor of the inflammatory cascade, exhibits an expression level positively correlated with the severity of depression (76). This cytokine additionally enhances the expression of other pro-inflammatory factors by activating the NF-κB signaling pathway, thereby establishing a vicious inflammatory cycle (77). Natural polysaccharides can effectively inhibit TNF-α production by modulating immune cell function and disrupting TLR4/NF-κB signal transduction (78). The synchronous downregulation of IL-1β merits consideration, as it can directly affect neuronal excitability and synaptic plasticity, playing a pivotal role in neuroinflammation (79). The persistent inhibition of these two key inflammatory factors indicates that natural polysaccharides may influence the upstream regulatory nodes of the inflammatory response (80, 81). Natural polysaccharides exhibit differential regulatory effects on IL-6 (82). Meanwhile, the increase in the anti-inflammatory cytokine IL-10 further suggested the ability of natural polysaccharides to restore immune balance.

Moreover, a previous study reported a simultaneous decline in NLRP3, caspase-1, and IL-18, suggesting that the intervention measures may specifically target the inflammasome pathway (83). As an intracellular sensor of danger signals, NLRP3, upon activation under stress conditions, recruits ASC and activates caspase-1, which may promote the maturation and release of IL-1β and IL-18 (84). Preclinical evidence suggests that NLRP3 knockout mice exhibit increased stress resistance, and pharmacological inhibition of NLRP3 has been reported to produce behavioral effects comparable to conventional antidepressants (85). Changes in NF-κB signaling exhibited heterogeneity across studies. Under basal conditions, moderate NF-κB inhibition may reduce transcription of pro-inflammatory genes, potentially alleviating inflammation (86). However, in specific brain regions, transient NF-κB activation may regulate neurotrophic factor expression and synaptic plasticity (87). A decline of MCP-1 suggests reduced microglial chemotaxis and aggregation in the central nervous system (88). Combined increases in TGF-β and IL-17 and decreased IL-22 expression may indicate a shift toward a regulatory T cell(Treg) phenotype, with relative inhibition of the Th17 pathway (89). Th17/Treg imbalance correlates with depression severity (90). A dominant Treg state supports immune tolerance and limits the propagation of central inflammation. The decrease in IL-22 may reflect the adaptive immune regulation related to the intestinal barrier function, consistent with observed remission of intestinal inflammation (91). Collectively, these findings indicate that the antidepressant effect of natural polysaccharides may be closely associated with multi-level immune regulation, from the intestinal microenvironment to the systemic circulatory system, influencing the immune status of the central nervous system.

### 4.4 Antioxidant effects

Relevant studies have suggested that the activities of T-SOD and CAT increased, whereas MDA and H_2_O_2_ levels decreased. SOD is the first line of defense against oxidative stress, facilitating the conversion of superoxide anion(O_2_•^-^) into H_2_O_2_ (92). CAT catalytically decomposes H_2_O_2_ into harmless water and oxygen (93). The simultaneous increase in the activities of these two key antioxidant enzymes suggests that natural polysaccharides may enhance the body’s inherent capacity to clear ROS, potentially preventing the accumulation and subsequent chain reaction. Additionally, MDA is the end product of lipid peroxidation, and its concentration reflects the severity of oxidative damage to the cell membrane structure (94). The decrease in MDA is molecular evidence suggesting that oxidative damage may have been reduced, which may indicate enhanced protection of neuronal and somatic cell membrane integrity (95).

A study reported that Keap1 protein was downregulated, accompanied by increased n-Nrf2 levels and decreased c-Nrf2 levels. Nrf2 is the “master switch” of cellular antioxidant responses (96). The observation of Keap1 downregulation and Nrf2 nuclear translocation suggests that natural polysaccharides may not only act as exogenous antioxidants but may also influence the endogenous antioxidant defense system, potentially leading to its upregulation (97). Regarding depression, alleviating oxidative stress may have important neuroprotective implications. Chronic stress and neuroinflammation may contribute to dysfunction of the mitochondrial electron transport chain, potentially leading to excessive production of ROS (98). Excessive ROS may damage lipids, proteins, and DNA of neurons, induce synaptic plasticity damage, form a vicious cycle with neuroinflammation, and potentially lead to neuronal dysfunction or death (99). It has been suggested that natural polysaccharides may disrupt this cycle through activation of the Nrf2 pathway and enhancement of SOD and CAT activities. This may result in improvement of molecular indicators and protection of neurons in emotion-related brain regions, including the hippocampus and prefrontal cortex. Natural polysaccharides may offer certain advantages compared with conventional antidepressants, as they enhance endogenous antioxidant defense in a systematic manner and may regulate the Nrf2/Keap1 signaling pathway, and may therefore target oxidative stress mechanisms associated with depression.

### 4.5 Gut-brain axis regulation

#### 4.5.1 Intestinal barrier integrity

Although only a limited number of studies have directly reported intestinal barrier indicators, the observed data suggest a trend. Natural polysaccharides were reported to upregulate essential tight junction proteins in colon and small intestine tissues, including ZO-1, occludin, and claudin-1. Tight junctions are multiprotein complexes that preserve the selective barrier function between intestinal epithelial cells (100). ZO-1 is an important cytoplasmic scaffold protein that anchors transmembrane proteins (including the occludin and claudin families) to the cytoskeleton, collectively establishing a dynamic “gating” system (101). Chronic stress conditions typically result in the downregulation of these proteins, causing compromised intestinal barrier function and the development of “leaky gut” (102). The upregulation of these key proteins suggests that natural polysaccharides may enhance intestinal epithelial defense and help restore normal physical barrier function. Studies have reported that natural polysaccharides were associated with reinstatement of gut barrier integrity, accompanied by increased expression of tight junction proteins. This may reduce LPS translocation, potentially decreasing a source of systemic and neuroinflammation. The decrease in serum LPS level observed in the previous discussion correlates with the increase in protein levels, such as ZO-1.

#### 4.5.2 Interaction between gut barrier and HPA axis

There may be a reciprocal regulation between intestinal barrier function and HPA axis activity. Elevation of LPS levels in the circulation is an activator of the HPA axis (103). Therefore, by restoring the intestinal barrier and diminishing LPS translocation, natural polysaccharides may indirectly mitigate the excessive activation of the HPA axis, consistent with the findings of reduced serum CORT and ACTH levels (104). An improved intestinal environment may lead to reduced systemic stress signals, potentially aiding in the normalization of the brain’s stress response system. The upregulation of intestinal tight junction proteins (ZO-1, occludin, and claudin-1) may be an early event contributing to the antidepressant-like effects of natural polysaccharides. It may provide a structural basis for “microbiota-gut-brain axis” communication.

#### 4.5.3 Gut microbiota diversity

Natural polysaccharide intervention was associated with substantial and complex regulatory effects on the intestinal microecology of depression model animals. The α-diversity index indicates the richness (Chao1; ACE index) and uniformity(Shannon; Simpson index) within the intestinal microbiota community (105). In most studies, treatment with natural polysaccharides was associated with improvements in the richness and uniformity of the microbiota. Chronic stress models typically result in a reduction in gut microbiota diversity (106). The overall increase in Chao1 and Shannon indices after polysaccharide intervention suggested that it may help n counteract microbiota depletion induced by stress, potentially augment diversity, and enhance the balance among various bacterial species. A highly diverse intestinal ecosystem can resist external disturbances, which is important for maintaining the host’s physiological homeostasis (107). Under certain pathological conditions, polysaccharides may be associated with a transient decrease in abundance, possibly due to selective inhibition of excessive proliferation of certain opportunistic pathogenic bacteria, which may reflect the ecosystem’s restoration toward a healthier state. Moreover, the Simpson index has shown inconsistent trends across studies, suggesting that the effects of polysaccharides vary among different microbial taxa. The primary effect appeared to be the establishment of functional homeostasis rather than a mere numerical increase in microbial abundance.

β-Diversity(indicated through analyses, including PCoA, NMDS, and PCA) evaluates the variations in the microbial community structure of samples among different groups, which is an important criterion for measuring whether intervention measures can reverse dysbiosis (108). The analysis of β-diversity indicated a consistent pattern. The microbiota composition of the polysaccharide treatment group differed from that of the depression model group and exhibited a clear trend of aggregation or convergence compared with the healthy control group. The observed changes were not limited to altering specific bacteria; rather, they were associated with comprehensive restoration of the disordered intestinal microbiota ecosystem toward a healthier state. Modulation of gut microbiota diversity may be one of the mechanisms by which natural polysaccharides are associated with antidepressant-like effects. This was associated with a shift from an imbalanced and pro-inflammatory intestinal environment toward a more balanced and protective state, accompanied by increased richness and stability of the ecosystem and a return of its overall structure toward that of healthy controls.

#### 4.5.4 F/B ratio

Conventionally, a higher F/B ratio is frequently associated with obesity and high energy consumption (109). The Firmicutes are believed to be more proficient in extracting energy from food, enhancing SCFA production, and increasing the energy harvest of the host (110). The Bacteroidetes phylum is associated with the utilization of host-derived carbohydrates (111). In depression, chronic stress often leads to metabolic disorders, but it is not simply manifested as obesity or emaciation (112). Therefore, the variation in the F/B ratio may depend on the specific metabolic phenotype, dietary structure, and pathological stage of the depression model (113). Restorative changes in Firmicutes may be important for reconstructing intestinal SCFA levels, enhancing intestinal barrier integrity, and providing neuroprotection. In many cases, these changes were accompanied by increases in SCFA levels, enhanced intestinal barrier function, and reduced systemic inflammation. These observations suggest that natural polysaccharides may simultaneously enhance the function of the gut-brain axis through multiple microecological pathways.

#### 4.5.5 SCFAs

Analysis of the levels of SCFAs in multiple studies suggested that natural polysaccharide intervention may be associated with increased levels of most SCFAs in animal models of depression. The increase in SCFAs has been proposed as one of the potential mechanisms by which natural polysaccharides might contribute to systemic effects. The report indicated that the three core SCFAs-acetic acid, propionic acid, and butyric acid-showed an upward trend in most studies. These SCFAs are the main end products of dietary fiber fermentation by intestinal microorganisms. The general increase in their levels provides preliminary evidence that natural polysaccharides may be efficiently metabolized by specific gut microbiota and converted into biologically active signal molecules. The increase in butyric acid is particularly notable among all SCFAs. It is the preferred energy source for colonic epithelial cells, and the increase in its level was reported to be associated with upregulation of intestinal barrier proteins (114).

Butyric acid enhances the repair of intestinal barrier function by providing energy to intestinal epithelial cells, establishing an associated with relationship with the previously observed reduction in serum LPS levels (115). In the brain, it upregulates the expression of genes, including BDNF, through epigenetic mechanisms, thereby facilitating neurogenesis, synaptic plasticity, and neuronal survival (116). Acetic acid and propionic acid play complex and crucial functions in regulating energy metabolism and immunity throughout the body (117). Acetic acid is the predominant SCFA in the peripheral circulation, capable of crossing the blood-brain barrier and influencing appetite regulation and energy balance in the hypothalamus (118). Propionic acid plays a dual role; on the periphery, it has powerful immunomodulatory and anti-inflammatory properties, which may help suppress systemic inflammation (119). However, excessively elevated levels of propionic acid potentially have complex effects on the central nervous system (120). Herein, the synergistic increase of propionic acid with acetic acid and butyric acid is more likely to point to a beneficial and balanced metabolic output pattern, potentially contributing to the homeostasis of the metabolism-immune-nervous system.

Branched-chain SCFAs, including isobutyric acid and isovaleric acid, primarily originate from protein fermentation (121). Their changes may more accurately reflect the regulation of the microbiota function by natural polysaccharides: enhancing carbohydrate fermentation and relatively inhibiting the putrefactive fermentation of proteins. This shift in metabolic pathways may reduce the synthesis of harmful metabolites such as ammonia and phenols, which could enhance the intestinal environment. The increase of hexanoic acid signifies the enrichment of a specific bacterial community capable of synthesizing longer-chain fatty acids, potentially enriching of the microbial metabolic library. Some individual studies have reported a decline in SCFAs levels, which does not diminish the importance of SCFAs but rather highlights the high complexity and context-dependent nature of microbiota metabolism. This phenomenon may pertain to variations in intervention duration, polysaccharide type, baseline microbiota structure, or the host’s absorption and utilization capabilities. These abnormal data indicate that SCFA production is a dynamic equilibrium process, with its final level resulting from the interplay of synthesis, absorption, and host metabolism.

#### 4.5.6 Phylum and genus-level changes

An integrated analysis of microbiota changes at the phylum and genus levels across various studies has suggested that the regulation of the intestinal microecology in animal models of depression by natural polysaccharides may exhibits a highly complex but targeted “rebalancing” pattern.

At the phylum level, the extensive enrichment of the Bacteroidota phylum was a commonly observed findings (122). As the main degraders of complex polysaccharides and dietary fiber, the increase in the abundance of Bacteroidetes may reflect effective utilization of natural polysaccharides. The proliferation of this bacterium suggests an increase in SCFA production, especially acetic acid and propionic acid, which provide energy for the host and are also essential for maintaining the integrity of the intestinal barrier and regulating immune homeostasis (123). An increase in its ratio to F/B is frequently associated with a healthier metabolic phenotype. The extensive decline of the Proteobacteria phylum is a positive signal. This phylum contains various Gram-negative pathogenic bacteria(*Escherichia coli* and *Salmonella*) and is the primary source of endotoxin LPS (124). A decrease in its abundance may indicate reduced inflammatory signals from the intestine, which is consistent with the observed decrease in serum inflammatory factor levels and the alleviation of HPA axis hyperactivity. Similarly, the reduction of Campylobacterota was associated with an anti-inflammatory microenvironment (125). Overall, polysaccharide intervention facilitates the “survival of the fittest” within the *Firmicutes* phylum; it promotes the growth of beneficial members and inhibits harmful members.

At the genus level, natural polysaccharides enhanced regulatory capabilities. Genera including *Roseburia*, *Turicibacter*, and *Faecalibaculum* are all important butyrate producers (126). Butyrate serves as the primary energy substrate for colonic epithelial cells and is essential in strengthening the intestinal barrier, reducing the translocation of LPS into the bloodstream, and providing systemic anti-inflammatory and neuroprotective benefits (127). Their upregulation has been suggested as a potential link through which polysaccharides may exert effects. The increase in the genera *Bifidobacterium* and *Lactobacillus* in most studies has suggested the prebiotic effect of polysaccharides (128). These strains can produce beneficial metabolites such as lactic acid and acetic acid, inhibit pathogenic bacteria through competitive rejection, and directly engage in gut-brain axis communication by modulating immunity and generating neuroactive substances (129). The reduction of opportunistic pathogenic bacteria, including *Helicobacter* and *Klebsiella*, directly mitigates the immune stress and inflammatory risk of the intestinal mucosa (130). Some genera of bacteria, including *Clostridium*, which may generate harmful metabolites, are suppressed, thereby enhancing the chemical environment of the intestinal tract (131). The therapeutic effects of natural polysaccharides at the phylum level were characterized by the enrichment of Bacteroidetes (a phylum with strong polysaccharide-degrading and metabolic abilities) and the suppression of Proteobacteria (a phylum abundant in pathogens). This rebalancing was associated with a shift toward functions that may support anti-inflammatory and metabolic health. At the genus level, the changes included enhancement of butyrate-producing bacteria and classic probiotics, as well as reduction of potentially pathogenic flora, suggesting a possible link to systemic effects.

### 4.6 Limitations and future directions

In the 20 animal studies included in this review, risk of bias was assessed using the SYRCLE tool. The results indicated that most studies reported key methodological domains incompletely. These widespread “unclear risk” ratings have important implications for the robustness of our conclusions. First, insufficient reporting of randomization and allocation concealment raises the concern of selection bias; if the groups were not truly comparable at baseline, the estimated antidepressant-like effects could be confounded. Indeed, five studies did not clearly report baseline characteristics, which not only reduces confidence in between-group comparability but also raises the possibility that the observed behavioral improvements may have arisen from pre-existing group differences rather than from the intervention itself. Second, the behavioral tests commonly employed in these studies (e.g., sucrose preference test, forced swim test, and tail suspension test) are susceptible to subjective influence. Because the blinding of caregivers and outcome assessors was not clearly documented, performance bias and detection bias cannot be excluded, and such biases tend to inflate the apparent treatment effects. It should be noted that an “unclear risk” rating does not confirm the presence of bias, but it reflects insufficient reporting transparency, making it difficult for readers to judge whether bias has been adequately controlled. Therefore, although the available results suggest that natural polysaccharides may alleviate depressive-like behaviors, the high proportion of “unclear risk” ratings across multiple domains limits the internal validity and certainty of the current evidence. These findings should be considered promising preclinical signals rather than definitive evidence of efficacy.

Another limitation is that this review is based entirely on animal models. Although models such as chronic unpredictable mild stress or corticosterone injection can recapitulate certain depression-like features, rodents differ from humans in gut microbial composition, neuro-immune-endocrine regulation, disease heterogeneity, and psychosocial context. Moreover, the polysaccharide preparations used in animal studies are often highly purified, and the doses and treatment durations may not be consistent with clinical conditions. The mechanisms described herein are therefore best interpreted as preclinical leads.

To strengthen the quality of future evidence, we recommend that animal experiments strictly adhere to the ARRIVE guidelines and fully report the randomization method, allocation concealment, blinding of caregivers, operators, and outcome assessors, as well as sample size estimation. Meanwhile, systematic dose-response studies and long-term safety evaluations are warranted, along with the development of more clinically relevant animal models-for instance, composite models that incorporate genetic susceptibility, chronic stress, and gut microbiota alterations. Ultimately, rigorous randomized controlled trials using standardized polysaccharide extracts are needed to assess their safety and efficacy as adjunctive treatments for depression. These efforts are essential for progressively translating basic research on natural polysaccharides into practical strategies for depression management.

## 5 Conclusion

This study systematically evaluated the antidepressant potential of natural polysaccharides by analyzing 20 preclinical studies. The results suggest that natural polysaccharides may ameliorate depressive-like behaviors, modulate monoamine neurotransmitter levels, and be associated with reduced neuroinflammation and oxidative stress. Mechanistically, these effects appear to be closely associated with reprogramming of the gut microbiota, including optimization of microbial community structure, enhancement of intestinal barrier function, and regulation of SCFAs metabolism, suggesting a potential role of the microbiota-gut-brain axis. However, the current evidence shows only a coexistence of polysaccharide-induced microbiota changes and behavioral improvements, and does not establish a causal pathway along the microbiota-gut-brain axis. Definitive validation will require further studies using germ-free animals or fecal microbiota transplantation. Furthermore, these findings should be interpreted with caution, as the analysis is based solely on animal models, and substantial heterogeneity exists in polysaccharide sources, dosages, and treatment durations across studies. The current preclinical evidence suggests the promise of natural polysaccharides, but it does not yet establish their clinical efficacy and safety. Future well-designed clinical trials, together with in-depth mechanistic studies identifying specific microbial strains and metabolites, are needed to determine whether these polysaccharides can be translated into effective interventions for depression.

## Data Availability

The original contributions presented in the study are included in the article/**Supplementary Material**. Further inquiries can be directed to the corresponding author.
