# Supplementary material for: Gut microbiota-dependent mechanisms and efficacy of natural polysaccharides in multitarget antidepressant therapy: a systematic review
Source: Front Psychiatry. 2026 Jun 29;17:1789107. doi: 10.3389/fpsyt.2026.1789107 (PMC13357710; doi:10.3389/fpsyt.2026.1789107)
Supplement: Supplementary file 1 [file SupplementaryFile1.doc]

**Literature search strategy**

1. **PubMed Search**

| **NO** | **Search Details** | **Results** |
| --- | --- | --- |
| #1 | (("Depression"[Mesh]) OR ("Depressive Disorder"[Mesh])) OR ((((((((((((((((((((((((((((((Depressive Symptoms[Title/Abstract]) OR (Depressive Symptom[Title/Abstract])) OR (Symptom, Depressive[Title/Abstract])) OR (Emotional Depression[Title/Abstract])) OR (Depression, Emotional[Title/Abstract])) OR (Depressive Disorders[Title/Abstract])) OR (Disorder, Depressive[Title/Abstract])) OR (Disorders, Depressive[Title/Abstract])) OR (Neurosis, Depressive[Title/Abstract])) OR (Depressive Neuroses[Title/Abstract])) OR (Depressive Neurosis[Title/Abstract])) OR (Neuroses, Depressive[Title/Abstract])) OR (Depression, Endogenous[Title/Abstract])) OR (Depressions, Endogenous[Title/Abstract])) OR (Endogenous Depression[Title/Abstract])) OR (Endogenous Depressions[Title/Abstract])) OR (Melancholia[Title/Abstract])) OR (Melancholias[Title/Abstract])) OR (Unipolar Depression[Title/Abstract])) OR (Depression, Unipolar[Title/Abstract])) OR (Depressions, Unipolar[Title/Abstract])) OR (Unipolar Depressions[Title/Abstract])) OR (Depressive Syndrome[Title/Abstract])) OR (Depressive Syndromes[Title/Abstract])) OR (Syndrome, Depressive[Title/Abstract])) OR (Syndromes, Depressive[Title/Abstract])) OR (Depression, Neurotic[Title/Abstract])) OR (Depressions, Neurotic[Title/Abstract])) OR (Neurotic Depression[Title/Abstract])) OR (Neurotic Depressions[Title/Abstract])) | 321559 |
| #2 | ("Gastrointestinal Microbiome"[Mesh]) OR ((intestinal flora) OR (gut microbiota) OR (intestinal microflora) OR (gut microbiome) OR (intestinal bacteria) OR (intestinal micro ecology) OR (gut microbial ecology) OR (gut micro ecology)) | 206674 |
| #3 | ("Polysaccharides"[Mesh]) OR ((polysaccharides) OR (saccharides) OR (glycan) OR (Oligosaccharide) OR (carbohydrate) OR (saccharides) OR (coarse polysaccharide) OR (pectic polysaccharide depolymerization) OR (pectin) OR (pectine) OR (microcrystalline cellulose) OR (fibrin) OR (cellulin) OR (cellulose) OR (starch) OR (raw starch) OR (corn starch) OR (starch flour) OR (starch content) OR (Amylum Starch) OR (amidon)) | 2851387 |
| #4 | #1 AND #2 AND #3 | 246 |

1. **Web of science Search**

| **NO** | **Search Details** | **Results** |
| --- | --- | --- |
| #1 | (TS=(( "Depressive Symptoms" OR "Depressive Symptom" OR "Emotional Depression" OR "Depressive Disorders" OR "Depressive Disorder" OR "Depressive Neurosis" OR "Depressive Neuroses" OR "Endogenous Depression" OR Melancholia OR "Unipolar Depression" OR "Depressive Syndrome" OR "Neurotic Depression" ) )) | 1516**11** |
| #2 | **(TS=( ("gut microbio*" OR "intestinal flora" OR "gut flora" OR "intestinal microbio*" OR "intestinal bacteria" OR "gut microflora" OR "intestinal microflora") OR ("gut micro ecology" OR "gut microbial ecology" OR "intestinal micro ecology")))** | **134797** |
| #3 | **(TS=(polysaccharide* OR glycan* OR oligosaccharide* OR carbohydrate* OR pectin OR cellulose OR starch OR amylum OR amidon)) OR (TI=("coarse polysaccharide" OR "pectic polysaccharide depolymerization" OR "microcrystalline cellulose" OR "raw starch" OR "corn starch" OR "starch flour" OR "starch content"))** | **578225** |
| #4 | #1 AND #2 AND #3 | 44 |

1. **Embase Search**

| **NO** | **Search Details** | **Results** |
| --- | --- | --- |
| #1 | 'depression'/exp OR 'depression' OR 'melancholia'/exp OR 'melancholia' OR 'endogenous depression'/exp OR 'endogenous depression' OR 'unipolar depression'/exp OR 'unipolar depression' OR 'eurotic depression'/exp OR 'neurotic depression' OR 'depressive symptom*':ti,ab,kw OR 'emotional depression':ti,ab,kw OR 'depressive neurosis':ti,ab,kw OR 'depressive neuroses':ti,ab,kw OR 'depressive syndrome':ti,ab,kw | 1104233 |
| #2 | 'intestinal microbiome'/exp OR 'gut flora'/exp OR 'gut microbio*' OR 'intestinal flora' OR 'intestinal microbio*' OR 'intestinal bacteria' OR 'gut microflora' OR 'intestinal microflora' OR 'gut micro ecology' OR 'gut microbial ecology' OR 'intestinal micro ecology' | 176122 |
| #3 | 'polysaccharide'/exp OR 'glycan'/exp OR 'oligosaccharide'/exp OR 'carbohydrate'/exp OR 'pectin'/exp OR 'cellulose'/exp OR 'starch'/exp OR 'amylum' OR 'amidon' OR 'coarse polysaccharide' OR 'pectic polysaccharide depolymerization' OR 'microcrystalline cellulose' OR 'raw starch' OR 'corn starch' OR 'starch flour' OR 'starch content' | 1342736 |
| #4 | #1 AND #2 AND #3 | 1145 |
